# EpipwR: efficient power analysis for EWAS with continuous outcomes

**DOI:** 10.1093/bioadv/vbaf150

**Published:** 2025-06-25

**Authors:** Jackson Barth, Austin W Reynolds

**Affiliations:** Department of Statistical Science, Baylor University, Waco, TX 76798, United States; Department of Microbiology, Immunology, and Genetics, University of North Texas Health Science Center, Fort Worth, TX 76107, United States

## Abstract

**Motivation:**

Epigenome-wide association studies (EWAS) have emerged as a popular way to investigate the pathophysiology of complex diseases and to assist in bridging the gap between genotypes and phenotypes. Despite the increasing popularity of EWAS, very few tools exist to aid researchers in power estimation and those are limited to case-control studies. The existence of user-friendly tools, expanding power calculation functionality to additional study designs, would be a significant aid to researchers planning EWAS.

**Results:**

We introduce EpipwR, an open-source R-package that can efficiently estimate power for EWAS with continuous or binary outcomes. EpipwR uses a quasi-simulated approach, meaning that data is generated only for CpG sites with methylation associated with the outcome, while *P*-values are generated directly for those with no association (when necessary). Like existing EWAS power calculators, reference datasets of empirical EWAS are used to guide the data generation process. Two numerical studies show the effect of the selected empirical dataset on the generated correlations and power, while another explores the accuracy of EpipwR against case-control alternatives. EpipwR is shown to outperform existing alternatives on both simulated and real EWAS datasets.

**Availability and implementation:**

The EpipwR R-package is currently available on Bioconductor or at github.com/jbarth216/EpipwR.

## 1 Introduction

Over the past decade, epigenome-wide association studies (EWAS) have emerged as the dominant way for researchers to investigate the relationship between epigenetic markers and phenotypes at the genome-wide level. DNA methylation has become the most widely studied epigenetic mechanism because of its ease to assay through existing microarray and sequencing technology, its ubiquity across the genome, and its impact on gene expression. DNA methylation, the process whereby a methyl group is added to a nucleotide changing its availability for transcription, occurs most often in the context of cytosine–phosphate–guanine (CpG) dinucleotides in millions of locations around the genome. To assay this variation at a population scale, most EWAS use microarray-based platforms for assessing DNA methylation, such as the Illumina Infinium HumanMethylation450 BeadChip (covering ∼450 000 CpG sites in the human genome) or the Illumina Infinium MethylationEPIC Array (covering >850 000 CpGs). Both arrays assess methylation at single-CpG resolution, quantified using a methylation β-value; an approximately continuously distributed measure that reflects the level of methylation at a specific locus, ranging from 0 (unmethylated) to 1 (methylated) (Bibikova *et al.* 2006). The development of these arrays has precipitated a huge interest in studying DNA methylation in the context of human health and disease (Rakyan *et al.* 2011, Murphy and Mill 2014, Wei *et al.* 2021). The need for power calculations to inform EWAS study design has often been reiterated since their advent (Michels *et al.* 2013, Tsai and Bell 2015, Mansell *et al.* 2019), but few tools have been created to aid researchers.

It is now widely accepted that formal power calculation and sample size justification is an essential part of genomic study design to ensure meaningful findings are reported. This has motivated the development of numerous statistical power evaluation tools for genome- and transcriptome-wide association studies, including the GAS Power Calculator (Johnson and Abecasis 2017), GWAPower (Feng *et al.* 2011), RnaSeqSampleSize (Zhao *et al.* 2018), RNAseqPS (Guo *et al.* 2014), and general tools such as OCplus, which calculates sample size for fixed cutoffs by optimizing for false discovery rates (Pawitan *et al.* 2005). However, surprisingly few tools for EWAS power evaluation have been developed, despite substantial work in the areas of DNA methylation data QC, normalization, and analysis (Houseman *et al.* 2012, Jaffe *et al.* 2012, Teschendorff *et al.* 2013, Aryee *et al.* 2014, Fortin *et al.* 2014, Morris *et al.* 2014, Peters *et al.* 2015, Teschendorff *et al.* 2017, Chakravarthy *et al.* 2018, Song and Kuan 2022). Although some sample size determination methods have been explored for cell-type and lineage-specific DNAm [see approaches used in You *et al.* (2020) and Zheng *et al.* (2018)], the pwrEWAS package (Graw *et al.* 2019) is perhaps the only tool currently available to researchers for easily estimating power under a variety of conditions (e.g. sample size, effect size, false discovery rate threshold). Even with the availability of this tool, most EWAS continue to be conducted without formal power analyses, resulting in potentially under- and over-powered studies (Tsai and Bell 2015). Furthermore, pwrEWAS is currently limited to case-control study designs, limiting the ability of researchers to easily estimate sample size requirements during study design.

The scope of EWAS, however, have expanded beyond two-group comparison designs into the realm of complex traits. For example, the relationship between age and CpG methylation has been extensively studied over the past decade (Hannum *et al.* 2013, Horvath 2013, Horvath and Raj 2018), with many applications to other health outcomes (Levine *et al.* 2018, Lu *et al.* 2019a,b, 2022, Belsky *et al.* 2020, 2022). More recently, EWAS have investigated CpG methylation correlated with a number of quantitative traits, including blood metabolites (Gomez-Alonso *et al.* 2021, Jhun *et al.* 2021), body mass (Aslibekyan *et al.* 2015, Demerath *et al.* 2015, Wahl *et al.* 2017), lung function (Herrera-Luis *et al.* 2022), and even behavioral health (Barbu *et al.* 2022, Montalvo-Ortiz *et al.* 2022, Raffington *et al.* 2024). As the number and complexity of phenotypes being studied with EWAS continues to grow, so does the research community’s need for appropriate and easily accessible power calculation tools.

Here, we introduce EpipwR, a publicly available tool for comprehensive EWAS power evaluation in the context of continuous or binary (e.g. case-control) outcome variables. EpipwR is a quasi-simulated approach to power analysis, in that data based on empirical EWAS are generated only for non-null distributions while false-positive *P*-values are generated directly using classic statistical theory. In this way, EpipwR leverages the unique form of EWAS data while running much faster than a fully simulated method. EpipwR was written using the R statistical programming language (R Core Team 2021) and the package is available at https://github.com/jbarth216/EpipwR.

## 2 Methods

Suppose an EWAS has *K* CpG sites and *n* distinct patient samples. Let $\beta_{ik}$ be the proportion of DNA methylation from patient *i*, CpG *k*, where it is assumed that $\beta_{ik}$ from a common CpG site are independently and identically distributed from some beta distribution, $\beta_{\cdot k}∼\mathrm{Beta}(\alpha_{k},\beta_{k})$. Of interest to researchers is a continuous phenotype $Y_{i}$ which is assumed to be correlated with the DNA methylation of some CpG sites, $\rho_{k}=\mathrm{cor}(Y,M_{\cdot k})$, where $M_{ik}=\mathrm{logit}(\beta_{ik})$. While $M_{ik}$ is usually closer to normality than $\beta_{ik}$ (Du *et al.* 2010), it should be noted that skewed distributions of $\beta_{ik}$ can still yield non-normal $M_{ik}$. Finally, *K* can be split into true nulls ($K_{n}$ of *K* have $\rho_{k}=0$) and false nulls ($K_{m}$ of *K* have $\rho_{k}\neq 0$). The goal of EpipwR is to evaluate the power of this analysis, defined as the proportion of false null hypotheses among $K_{m}$ that are correctly identified. The remainder of this section outlines and justifies the methodology implemented in EpipwR.

### 2.1 Data generation

**Figure 1.**
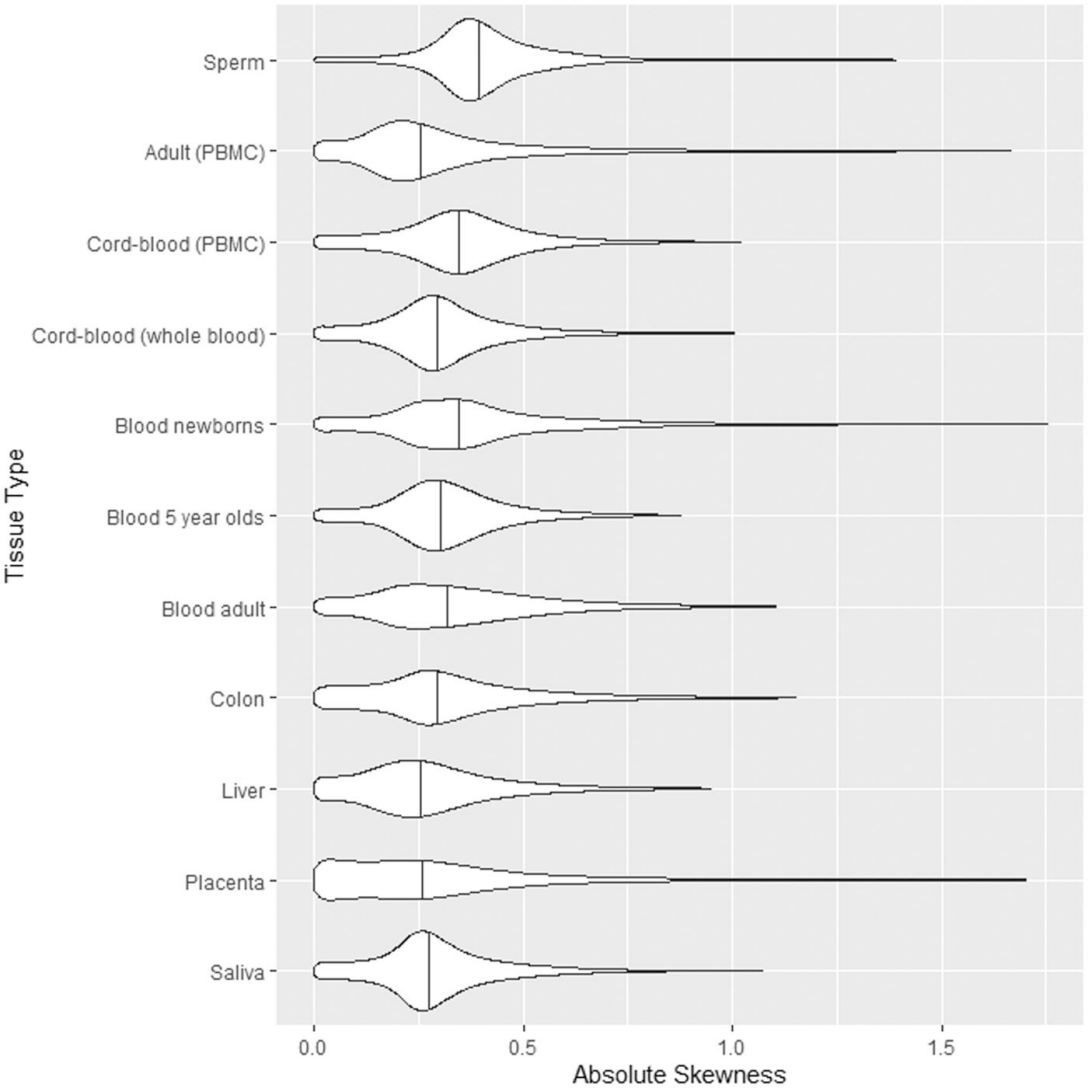
The violin plots show the distributions of absolute skewness across each of the 11 reference datasets used by EpipwR. For ease of viewing, the plots only show up to the 99th percentile of each dataset. The vertical lines display the median of each dataset.

The first step in calculating power for the scenario described above is to generate methylation data, $\beta_{ik}$. In an effort to maintain consistency across tools used in epigenomics, EpipwR applies the same method outlined by Graw *et al.* (2019) in pwrEWAS to generate the methylated proportions. To determine plausible distributions for each non-null CpG site (which will then be used to generate $\beta_{ik}$), EpipwR relies on empirical EWAS reference data. DNA methylation patterns have been shown to vary systematically across tissue types and disease states in the same tissue (Horvath 2013). To account for this variation in EpipwR’s power estimates, we include 11 reference datasets for users to choose from based on the relevance to the study being planned (Table 1 and Fig. 1). The datasets included are representative of the most common tissue types used in EWAS and were previously selected to maximize sample size of healthy subjects in these tissues (Graw *et al.* 2019). To provide maximum flexibility for users, we also include an option to input their own reference dataset for power calculations. This functionality will be useful for researchers who want to estimate power directly from preliminary data or those who are working in a tissue not included in the reference dataset. Once the dataset is selected/provided, EpipwR randomly selects one set of beta distribution parameters ${\hat{\alpha }}_{k}$, ${\hat{\beta }}_{k}$ for every CpG site assumed to have a non-null association with the phenotype. These parameter sets are determined through method of moments estimation on every CpG site in the selected dataset (for users providing their own datasets, EpipwR will calculate these parameter estimates as well). Unlike pwrEWAS, which is a fully simulated approach, methylation data are not generated for any CpG sites assumed to be “true nulls” in EpipwR (see section 2.2), which significantly reduces computation time and memory load.

**Table 1.** Reference datasets included in EpipwR, representing the most commonly used tissue types for EWAS.

Tissue	GEO Number	Samples limited to	Reference(s)
Adult (PBMC)	GSE67170	Disease state: control	Zhang *et al.* (2018)
Blood (Adults)	GSE42861	Subject: Normal	Kular *et al.* (2018), Liu *et al.* (2013)
Blood (Children)	GSE83334	Age: 5 years	Urdinguio *et al.* (2016)
Blood (Newborns)	GSE82273		Markunas *et al.* (2016)
Colon	GSE77718	Disease state: Normal	McInnes *et al.* (2017)
Cord-blood (PBMC)	GSE110128		Langie *et al.* (2018)
Cord-blood (whole blood)	GSE69176		N/A
Liver	GSE61258	Disease status: Control	Horvath *et al.* (2014)
Placenta	GSE62733	Health state: Normal	Kawai *et al.* (2015)
Saliva	GSE92767		Hong *et al.* (2017)
Sperm	GSE114753	Control	Jenkins *et al.* (2017)

Once the beta distributions have been identified, the next step is to generate *n* samples of $\left(Y,\beta_{\cdot 1},\beta_{\cdot 2},\ldots ,\beta_{.K_{m}}\right)$. EpipwR assumes that all $\beta_{ik}$ are conditionally independent on *Y* such that $\mathrm{Cov}(\beta_{\cdot k},\beta_{\cdot l}|Y)=0$ for the $k^{th}$ and $l^{th}$ CpG sites. This is a common simplifying assumption in power analysis, and one that removes the responsibility from the researcher of identifying a valid covariance structure for potentially thousands of $\beta_{\cdot k}$. This assumption allows *Y* to be sampled marginally, and then, the subsequent $\beta_{ik}|Y$ can be sampled concurrently. By default, EpipwR also assumes that *Y* is normally distributed and assumes without loss of generality that it follows a standard normal distribution. Therefore, EpipwR initially generates *n* samples of Y∼N(0,1). To conditionally sample $\beta_{ik}$, EpipwR utilizes a Gaussian copula. First, *n* samples of $X_{k}|Y$ are generated, where $X_{k}$ has a marginal standard normal distribution with $\rho_{k}=\mathrm{cor}(X_{k},Y)$. The percentiles of each $X_{ik}$ are then used to systematically generate $\beta_{ik}$, such that $\Phi (X_{ik})=F_{\beta_{\cdot k}}(\beta_{ik})$ where Φ is the standard normal cumulative distribution function (CDF) and $F_{\beta_{\cdot k}}$ is the CDF of $\beta_{ik}∼\mathrm{Beta}\left({\hat{\alpha }}_{k},{\hat{\beta }}_{k}\right)$ with parameters sampled from the empirical EWAS data. For example, suppose $x_{ik}$ is generated to be −0.8416. Since this is the 20th percentile value of the standard normal distribution, then $\beta_{ik}$ is assigned the 20th percentile value of its corresponding beta distribution. The $\beta_{ik}$ are then converted to $M_{ik}$ via the logit transformation. Since non-normal phenotype distributions can influence the power calculation, users also have the option to provide a sample of data from which to build generated data. If this is provided, the standard normal data is mapped to this distribution via Gaussian copula. See section 3.2 for alternative options.

In setting the target correlation $\rho_{k}$, researchers have the option of choosing a fixed correlation for all CpG sites such that $\mu_{\rho }=\rho_{k}$ or of identifying parameters of a truncated normal distribution to randomly generate the correlation of each associated CpG site:


$$\rho_{k}∼N(\mu_{p},\sigma_{\rho }),\;\rho \in \left\{\begin{array}{cc} \text{if}\;\mu_{\rho }>0, & \left[.03,1\right]\\ \text{if}\;\mu_{\rho }<0, & \left[-1,-.03\right] \end{array}\right\}.$$

For the non-null tests, $\rho_{k}$ cannot be in the range (−.03,.03), preserving practical significance.

### 2.2 Calculating *P*-values

For the $K_{m}$ associated CpG sites, the *P*-value calculations are standard. The sample correlation for each CpG site, $r_{k}$, is first calculated based on the chosen method (Pearson, Kendall, or Spearman) for each dataset, and then *P*-values are similarly determined. Since the nonparametric tests are rank-based, ties (i.e. two CpG sites having the exact same methylation level) in the data can throw off traditional estimation procedures. In the case of the Kendall or Spearman correlation tests, exact *P*-values are calculated only when no ties are present in the simulated data (this is often the case). Otherwise, the Pearson correlation is used as a replacement. Because EpipwR (like most power analysis software) does not take into account dependence between CpG sites unrelated to *Y*, using EpipwR with the Pearson test would yield the same results if the analysis was instead run using specialized software such as limma (Ritchie *et al.* 2015).

**Figure 2.**
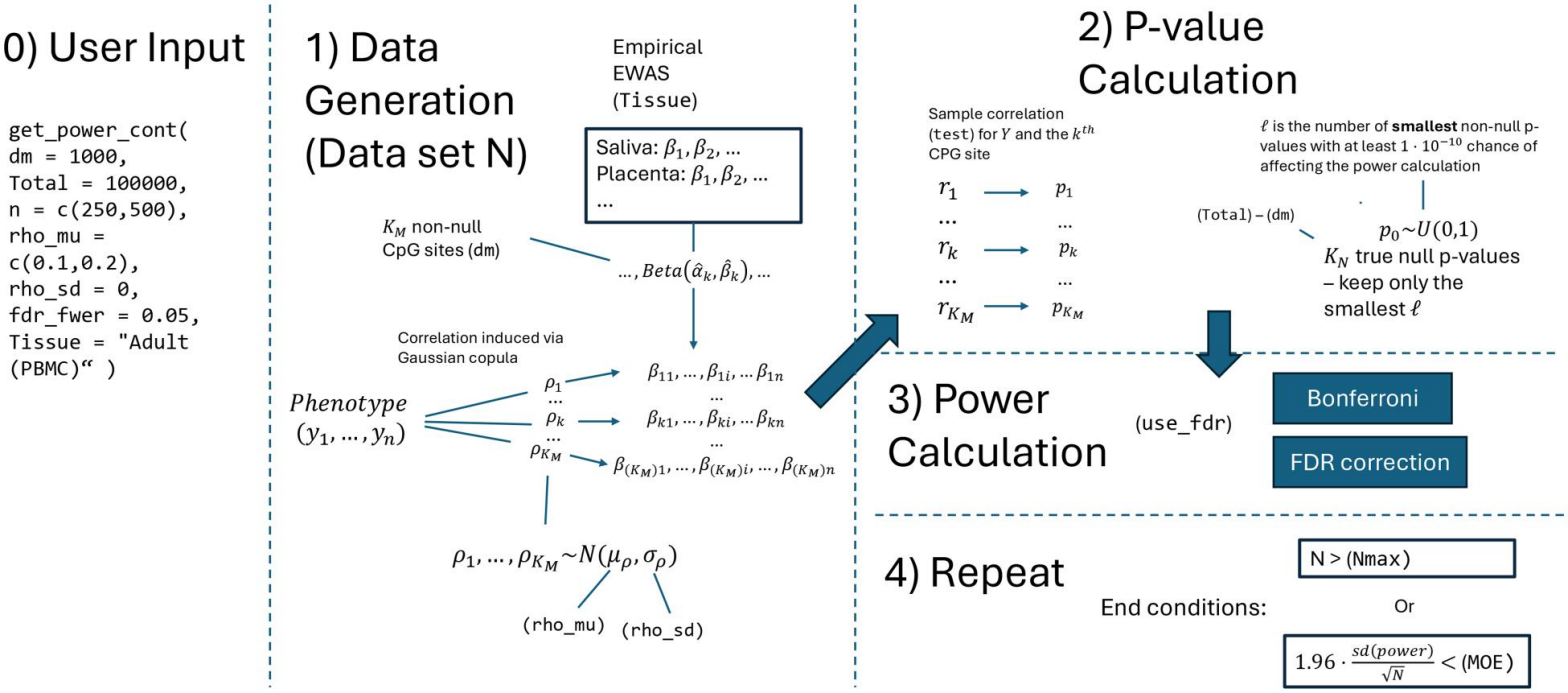
Diagram of full EpipwR workflow for continuous phenotypes.

If a Bonferroni adjustment is used to control for the family-wise Type I error rate, then *P*-values for the $K_{n}$ null tests can be safely ignored. If instead users wish to control for a false discovery rate (FDR), then additional *P*-values for the nonsignificant associations must be considered. For the $K_{n}$ CpG sites with a true null, computational resources are reduced significantly by generating *P*-values from a uniform distribution on the interval (0,1), a well-established result in statistical theory. However, even this approach can become computationally taxing if $K_{n}$ is large and the sampling is repeated on several datasets (N>100). To further improve computational efficiency, EpipwR then calculates the largest order statistic from the $K_{n}$  *P*-values with a probability of at least $1\times 10^{-10}$ of moving the cutoff and altering the power calculation (call this ℓ). For each dataset, EpipwR then draws only the first ℓ order statistics from $K_{m}$  U(0,1) samples, each of which can be sampled directly from a beta distribution (for a large number of tests, ℓ is around 30–40). All other *P*-values are assumed to be too large to alter the FDR cutoff. Figure 2 shows a diagram of all steps in the data generation and *P*-value calculation process.

### 2.3 Methodology for binary outcomes

When the outcome variable is binary, the data and *P*-value generation of EpipwR are similar to pwrEWAS (Graw *et al.* 2019) with a few key changes. First, while pwrEWAS ensures that the variance of each group is equivalent, EpipwR ensures that the precision of each group is the same (α+β for the beta distribution), which keeps the overall level of information balanced between each group. Next, EpipwR asks users to specify a mean and standard deviation for the non-null effect sizes and then generates them with a truncated normal distribution. In pwrEWAS, users are asked to specify the target maximal difference, and the function fits a distribution based on these values. This change was made for two reasons: (1) this ultimately gives the user more control over the characteristics of the effect sizes (including allowing all effect sizes to be equivalent) and (2) is easier to understand. Finally, like with continuous case, EpipwR only generates datasets for the non-null tests and uses a U(0,1) distribution to generate the null *P*-values.

### 2.4 Calculating power

EpipwR uses a traditional calculation to evaluate power, where power is the percentage of the $K_{m}$ truly associated CpG sites found to be significant. False positives are ignored in the power calculation; however, they can still impact the FDR cutoff by artificially raising it in very specific (and unlikely) circumstances. For instance, if the *P*-value for a true null test is lower than that of a non-null test, that raises the FDR threshold of the higher *P*-value test, making the power increase slightly. This calculation is repeated for each of the *N* datasets. The average across all *N* datasets is accepted as the average power.

Rather than specifying a specific number of datasets *N*, EpipwR uses a dynamic process to determine *N* as it runs. First, EpipwR calculates power for 20 different datasets (the minimum value for *N*). Beginning at the 20th dataset, it then calculates the margin of error of a 95% confidence interval for average power using the following formula,


(1) $$m_{\left(j\right)}=1.96\cdot \frac{s_{\left(j\right)}}{\sqrt{N_{\left(j\right)}}}$$

where $s_{\left(j\right)}$ is the sample standard deviation of power through the first $N_{\left(j\right)}$ generated datasets. If $m_{\left(j\right)}$ is less than a user-specified margin of error (the default is 0.03), the process stops and uses the results from the $N_{\left(j\right)}$ generated datasets to calculate average power. Otherwise, a new dataset is generated and the algorithm repeats, this time with $N_{\left(j+1\right)}=N_{\left(j\right)}+1$ datasets. The process continues until $m_{\left(j\right)}$ is small enough or if $N_{\left(j\right)}$ reaches a user-specified maximum (default is 1000) to limit the strain on computational resources.

### 2.5 EpipwR in practice

There are three main functions in the EpipwR package: two that calculate power for continuous and binary outcomes (get_power_cont and get_power_cc) and one that plots the results of the power analysis (EpipwR_plot). The inputs of get_power_cont and get_power_cc overlap quite a bit, as indicated in Supplementary Table S1, available as supplementary data at *Bioinformatics Advances* online. Since the sample size and $\mu_{\rho }$ inputs can take on multiple values, EpipwR calculates power separately for all scenarios under the Cartesian product of these two vectors. The output is a data frame with 1 row for every combination of n and rho_mu (or delta_mu) and columns tracking the average power, number of datasets used, and the standard error of the average power. EpipwR_plot takes this data frame and plots 95% confidence intervals using ggplot2 (Wickham 2016).

Supplementary Figure S1, available as supplementary data at *Bioinformatics Advances* online demonstrates the EpipwR workflow. In this scenario, researchers plan an EWAS with 100 000 CpG sites, of which 500 are expected to have non-null correlations with some phenotype of interest. The researchers wish to test sample sizes of 100–200 at denominations of 25 and $\mu_{\rho }\in (0.3,0.35,0.4)$, where $\rho_{k}$ are fixed at these values. At a 5% false discovery rate, the plots on the right-hand side of Supplementary Fig. S1, available as supplementary data at *Bioinformatics Advances* online show that sample sizes of 125 and 150 are high enough to achieve 80% and 90% power (respectively) if $\mu_{\rho }=0.4$, whereas larger sample sizes are needed for smaller correlations.

## 3 Results

In addition to the simulations and case study shown in this section, the Supplementary Material, available as supplementary data at *Bioinformatics Advances* online contains a direct comparison to pwrEWAS on computation time. For an overview of computational efficiency for continuous EpipwR, see Supplementary Table S2, available as supplementary data at *Bioinformatics Advances* online. For a direct comparison between computational efficiency of pwrEWAS versus case-control EpipwR, see Supplementary Table S3, available as supplementary data at *Bioinformatics Advances* online.

### 3.1 Impact of reference dataset on observed correlation and power

**Figure 3.**
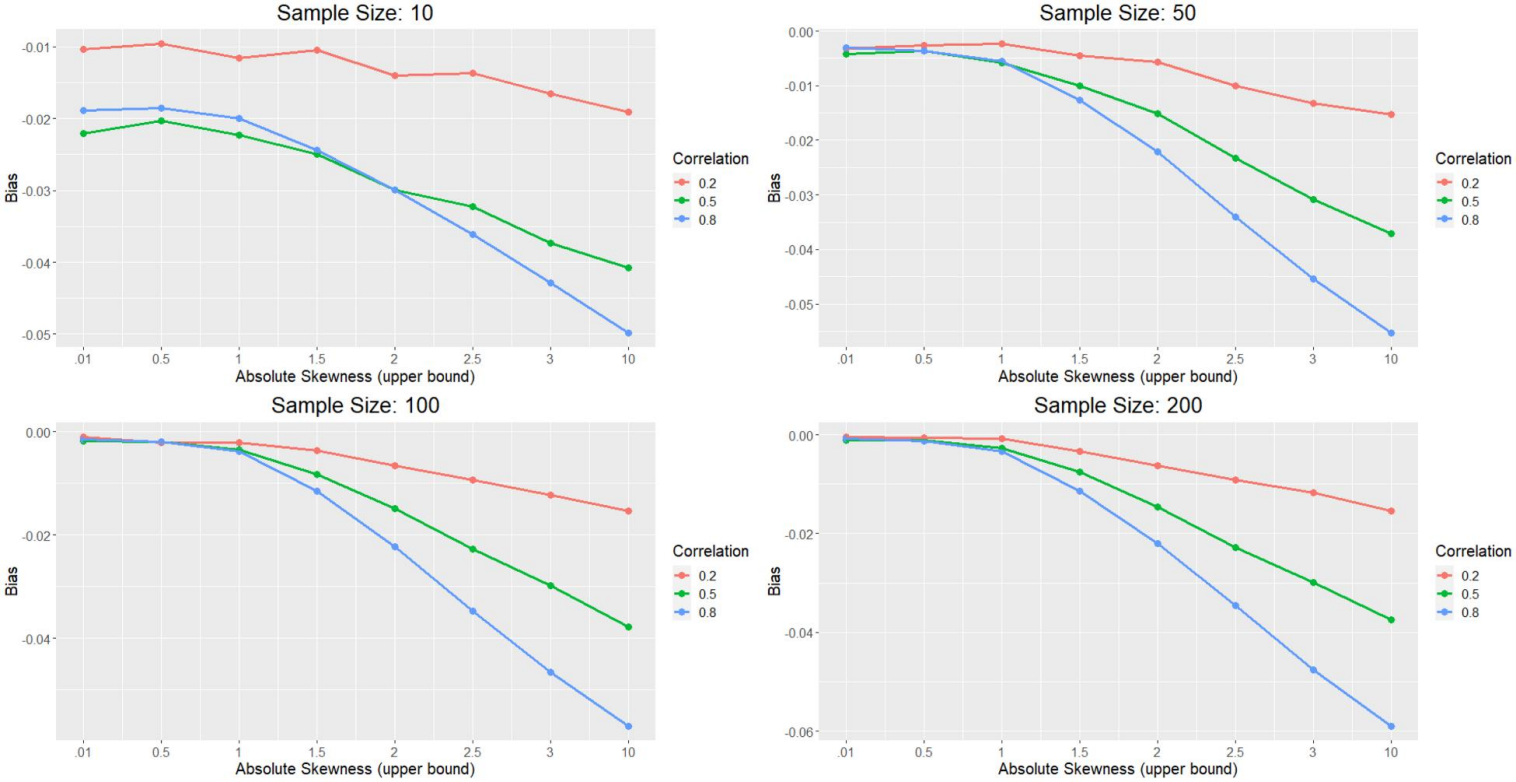
Correlation estimation results using a Gaussian copula. When skewness is low, there is very little bias in the correlation of the generated datasets. However, as absolute skewness grows, so does the bias, getting as low as −.06 for the most extreme scenarios.

To evaluate the efficacy of our method to produce dependent data, a small simulation was conducted to observe the large-sample behavior of the sample correlations under differing beta distributions. Sample datasets for CpG methylation were generated based on 800 $\hat{\alpha },\hat{\beta }$ estimates from the saliva dataset, drawn using stratified sampling based on the estimated skewness (γ) of the distribution. Specifically, 100 pairs of estimates were drawn such that |γ|∈(0,0.01), another 100 were drawn such that |γ|∈(0.01,0.5) and so on for a variety of ranges (see the *x*-axis of Fig. 3). Correlated data were then generated based on sample sizes n∈(10,30,50,100,150,200) and “true” correlation ρ from 0.1 to 0.9 in increments of 0.1. For each simulation setting, 1000 correlated datasets were generated based on the method described in section 2.1. The average and standard deviation of the sample correlations were then stored.

Figure 3 shows the results of this simulation. Unsurprisingly, the bias for each setting is always negative, confirming that this method tends to underestimate correlation. When the skewness of the beta distribution is low (γ<1), the bias is very close to 0, particularly for large sample sizes. As the skewness increases, the bias becomes more severe, reaching as high as −2%, −4%, and −6% for small, medium, and large correlations, respectively. It should be noted, however, that absolute skewness levels above 2 or 3 are very rare in the reference data (see Fig. 1)—0.61% and 0.14% frequency, respectively—while levels above 10 are nearly nonexistent (<.01% frequency). Therefore, average bias on the whole tends to be quite small.

Absolute skewness levels of each dataset are reported in Fig. 1. While the median of each dataset is well below 1, certain datasets (newborn blood and sperm) have a small amount of CpG sites with relatively high (>1) skewness levels. These distributions tend to generate slightly smaller linear correlations consistent with the results of the simulation, which in turn produce lower power levels. Given the rarity of these cases, the effect of these high-skewness distributions are marginal and tend to be washed out by the high frequency of distributions with low or moderate skewness.

**Figure 4.**
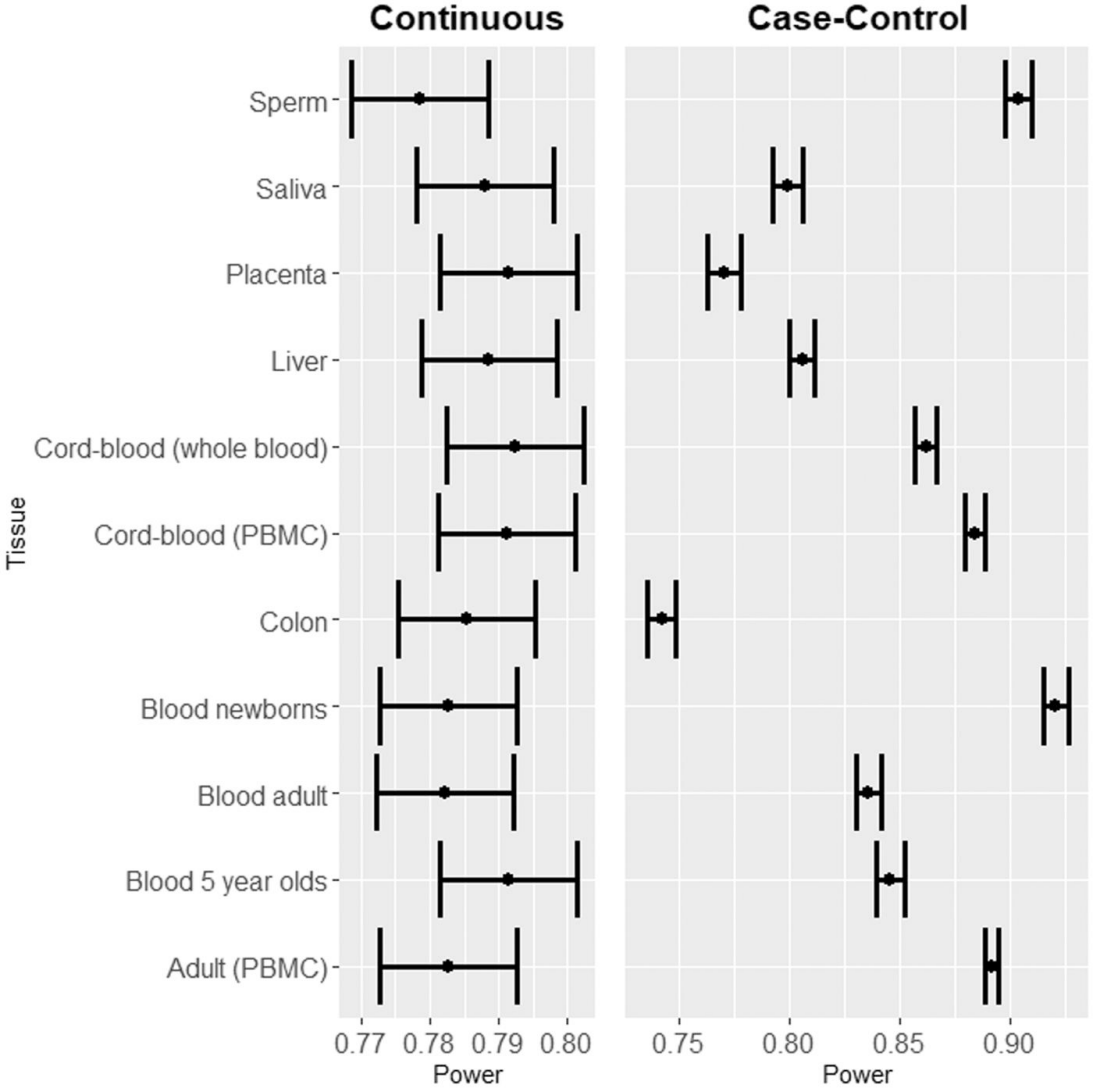
Power comparison by dataset for one setting (each). Continuous setting includes 1000 truly non-null tests, 100 000 total tests, sample size of 200, 5% FDR, and ρ=0.3. Case-control setting includes the same number of tests, sample size of 340, 5% FDR, and δ=0.02.

To test the impact of this on overall power, we ran EpipwR (continuous and case-control) on several different settings for all 11 tissue types. Figure 4 shows the calculated power and 95% confidence intervals of one setting of the continuous and case-control scenarios, as the results were consistent regardless of the setting. Based on these results, we find that the tissue selection has almost no impact when the phenotype is continuous but a very pronounced effect in the case-control scenario. This is likely due to the fact that a significance test for correlation is unaffected by a change in variance as long as the correlation remains fixed, but the same is not true for a difference in means. Furthermore, higher skewness datasets are more likely to produce values close to 0 or 1. When the logit transform is then applied, small mean differences will be further apart (i.e. a methylation difference of 0.01 on the logit scale is much larger for 0.98 and 0.99 then for 0.55 and 0.56, e.g.). For this reason, we recommend that researchers utilize the “custom” option if the available options are not close to the desired tissue type when using EpipwR for case-control analyses.

### 3.2 Simulation studies

In this section, we use a simulation study to evaluate the accuracy of EpipwR with continuous outcomes compared to a binarized case-control approach and when phenotype data is non-normal. The simulation settings considered included 400 000 and 800 000 total CpG sites, 10 and 100 differentially methylated CpG sites, and fixed correlations of 0.2 and 0.3. For each setting, sample sizes from 100 to 1000 in multiples of 100 were considered. Simulation data was generated by inducing correlation between a phenotype (*Y*) and the methylation levels at each differentially methylated CpG site via Gaussian copula. Distributions for *Y* included the following: normal data (generated in R), real data with heavier tails than a normal, and real data with moderate right skewness. Power was then calculated using FDR corrections. Each simulation setting was run 1000 times (or until the standard error was less than 0.01) to obtain ground truth levels of power. The phenotype and CpG data are from Houtepen *et al.* (2016), a multi-cohort study that observes the relationship between DNAm and Cortisol levels.

Although EpipwR is the first tool designed to specifically power continuous EWAS, it is possible to use case-control power calculators (such as pwrEWAS or case-control EpipwR) to estimate this calculation by finding some mean difference equivalent to the correlation. Using the test statistics for a Pearson correlation test and a two sample *t*-test, the mean difference that will produce the exact same test statistic has form $\delta =\frac{2\sigma_{x}r}{\sqrt{1-r^{2}}}\sqrt{\frac{n-2}{n}}$ (see Supplementary Material for the derivation, available as supplementary data at *Bioinformatics Advances* online), where $\sigma_{x}$ is the standard deviation of the untransformed data (in this case, the methylation data). Since $\sigma_{x}$ varies (in some cases, quite significantly) by CpG site, the transformation for simulation-based tools such as EpipwR and pwrEWAS is not straightforward. Because of this, we tested several potential transformations to a mean difference, including the 5th, 25th, 50th, 75th, and 95th percentiles of the logit-transformed standard deviations of the corresponding reference dataset used in the power calculation. Alternatively, tools such as OCplus (Pawitan *et al.* 2005), which are also designed for case-control analyses but use theoretical approximations, do not have this problem, since the standard deviation is specified by the user and can be set arbitrarily. For this reason, OCplus is also included as a point of comparison.

**Figure 5.**
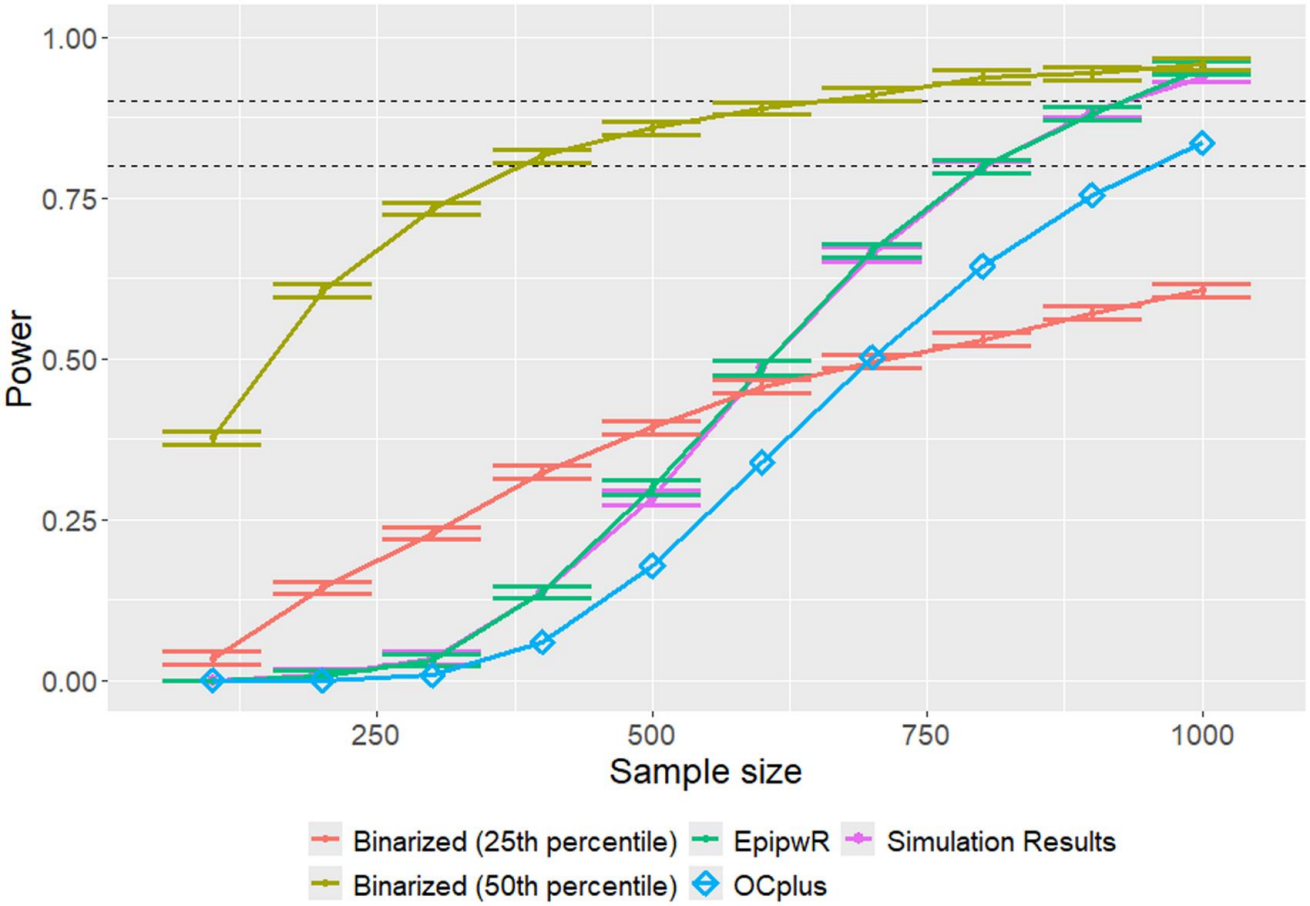
Power curves for EpipwR and binarized case-control power analysis against true (simulated) power. Specifically, this figure shows normal phenotype data with 10 truly associated CpG sites, 400 000 total CpG sites, and a fixed correlation of 20%.

Figure 5 shows the power curve of continuous EpipwR compared to the actual power, and the three case-control transformations for one setting (400 000 tests, 10 truly associated, ρ=0.2—see Supplementary File S2 for full results, available as supplementary data at *Bioinformatics Advances* online). In addition to EpipwR estimating power almost perfectly, it is clear from this figure that a case-control approach from a simulated tool cannot adequately replace continuous EpipwR. This is clearly indicated by the different shape of the curves. Because of this, there is no clear 1–1 way to map the correlation to a mean difference when the approach considers the variance heterogeneity present in the CpG sites, as EpipwR and pwrEWAS do. OCplus performs significantly better than the simulation-based binarized approaches, however consistently underestimates the power. This is likely because OCplus approximates the FDR without taking into account the total number of tests, only the proportion of null tests.

**Figure 6.**
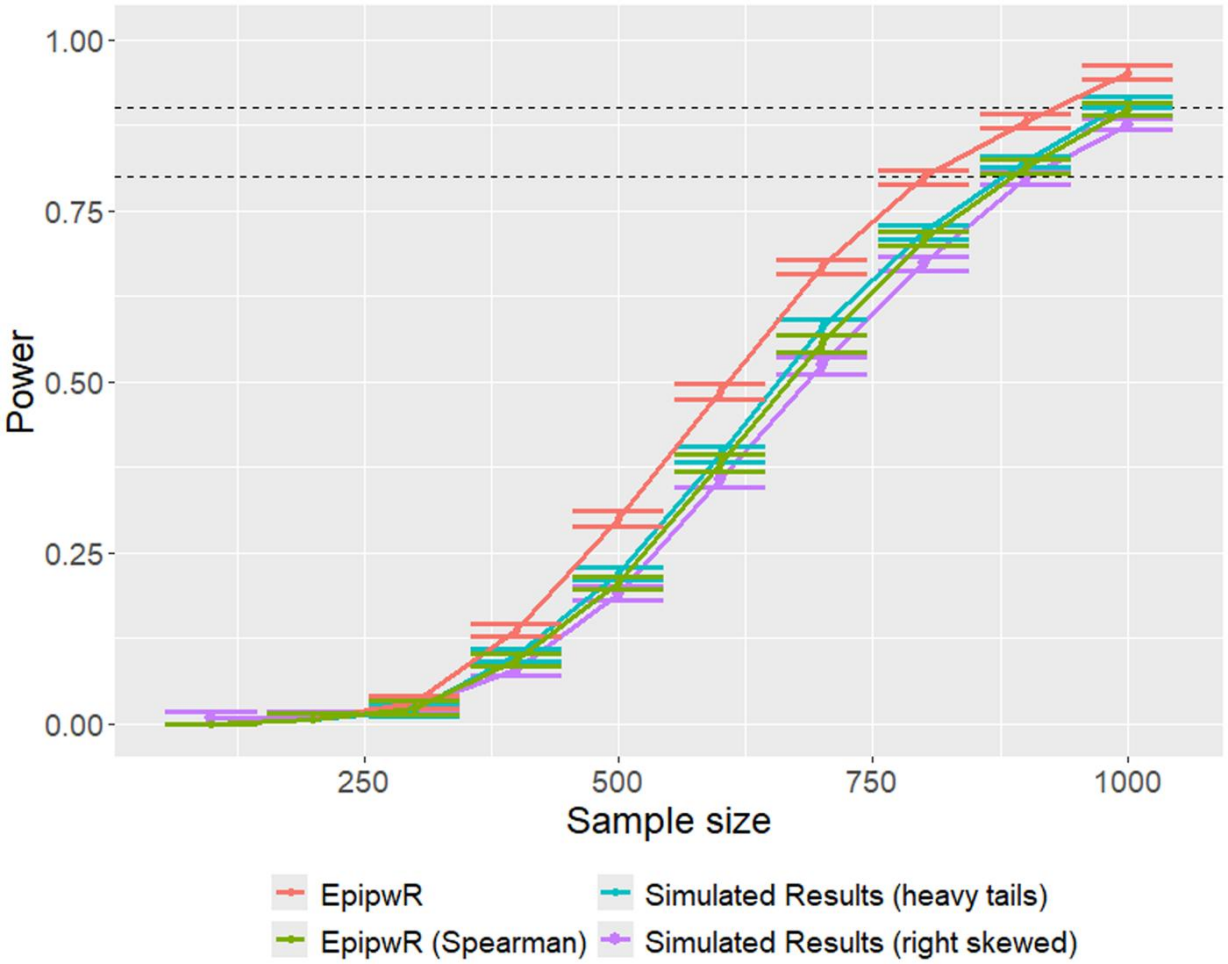
Power curves for EpipwR (Pearson and Spearman) against simulated power with non-normal phenotype distribution. The specific setting includes 10 truly associated CpG sites, 400 000 total CpG sites and a fixed correlation of 20%.

Figure 6 shows the power curves of EpipwR against simulated power with non-normal phenotype data. Unlike Fig. 5, EpipwR (with the Pearson correlation test) is a bit off the mark here, recommending smaller sample sizes than would actually be warranted. However, EpipwR tested using a rank-based correlation (Spearman, in this case), performs significantly better, with the power curve being directly on top of the heavy tail power values. This result is not surprising, as Spearman correlation is based solely on the order of the values. For this reason, we recommend that users with non-normal phenotype data use the nonparametric options for EpipwR. Alternatively, we have also included an optional argument where users can provide a sample of phenotype data that correlation will be mapped onto via the Gaussian copula.

Full results are included in Supplementary File S2, available as supplementary data at *Bioinformatics Advances* online, but they are very consistent with those shown in Figs 5 and 6.

### 3.3 Real data examples

Here, we provide three different case studies to examine the performance of EpipwR on real EWAS data and to demonstrate its use in practice.

#### 3.3.1 Case study: NIH stroke scale

This first case study is unique in that contains both a discovery and a replication cohort (with very little CpGs from the discovery replicating). Thus, it is a natural candidate to demonstrate the value of a power analysis tool such as EpipwR.

In a recent EWAS (Cullell *et al.* 2022), researchers studied the effect of DNA methylation on the NIH stroke scale (NIHSS), a score that determines the severity of symptoms experienced by stroke patients (Brott *et al.* 1989). Discovery was performed on a very large cohort (*n* = 643), whereas replication was performed on 44 CpG sites with the most significant associations with the NIHSS. A smaller cohort (*n* = 63) was used to replicate these findings for 38 of the CpG sites (the other 6 failed quality control), but only one was found to be significant at the p=.05 level. Although the replication cohort came from an already existing sample, EpipwR can help explain why the replication performed poorly. Table 2 in Cullell *et al.* (2022) indicates that the discovery cohort had 9 CpGs that were nominally significant ($p<1\cdot 10^{-6}$). Based on the reported summary statistics, we estimated the mean correlation to be 0.127 with a standard deviation of 0.014 (this includes some effect size shrinkage due to selection bias). EpipwR showed that a sample size of 62 would only have power to detect roughly 15% of the significantly associated CpG sites at the 5% significance level, which is consistent with the author’s expectations that the sample size of the replication study is a cause of the results (Cullell *et al.* 2022). A larger cohort of 200 would have power to detect about 44% of all truly associated CpG sites, but a cohort between 450–500 would be needed to see 80% of the nominally significant CpGs replicate.

**Table 2.** EpipwR and OCplus power calculations against average power from 10 000 over-sampled Cortisol datasets.^a^

*n*	% of truly significant tests rejected	EpipwR	OCplus
85	0.1000	0.0565	0.0053
100	0.1699	0.1153	0.0272
150	0.4979	0.4747	0.2844
200	0.7756	0.7939	0.6172
225	0.8641	0.8747	0.7403
250	0.9181	0.9415	0.8294

a EpipwR parameters were specified to have a maximum margin of error of 0.01.

#### 3.3.2 Oversampling with cortisol data

A relatively small EWAS (n=85) investigated the link between Cortisol levels and childhood trauma, using DNA methylation at certain CpG sites for mediation. As part of the analysis, correlation between cortisol and methylation was explored, with authors finding 3 CpG sites that were nominally significant ($p<1\cdot 10^{-6}$) but none significant after Bonferroni or FDR correction due to the small sample size and large number of tests (385,882). To further evaluate EpipwR, we compare power estimates for EpipwR at n=85 and larger sample sizes, using 10 000 datasets sampled with replacement from the cortisol data to evaluate the estimates. OCplus estimates are included as well for reference. Table 2 shows the results. Interestingly, both EpipwR and OCplus slightly underestimate power at low sample sizes. EpipwR becomes more accurate at larger sample sizes, while OCplus consistently underestimates power.

#### 3.3.3 Subsampling with BMI data

We also provide a similar analysis to the previous section, this time including a large EWAS (n=422) with BMI data (Zannas *et al.* 2019). The distribution of absolute-valued correlations between DNA methylation is bi-modal, with one peak at 0 and another around 0.24. Given that BMI is known to significantly correlate with methylation at several CpG sites, we interpret this bi-modality as a mixture of the null and non-null correlations. We estimated roughly 7000 CpG sites to be in the non-null distribution out of 453,351 in total and approximate the mean to be roughly 0.24 (the mode of the distribution) and assume a standard deviation of 0.02, which covers roughly 90% of the approximated distribution. For this study, we sampled from the EWAS at smaller sample sizes to evaluate power for both EpipwR and OCplus. We attempted to include the exact empirical distribution in OCplus by specifying all 7000 non-null correlations, but this was beyond the technical limitations of OCplus. Instead, we approximated the empirical distribution using quantiles. In this case, the true percentage of non-null tests that are found to be significant was estimated over 1000 datasets, given the larger number of non-null correlations (the standard error is about 1%). Table 3 shows similar results to the cortisol case study—EpipwR tends to underestimate power at small sample sizes, but becomes much more accurate once the power approaches “typical” levels (i.e. 70–90%). As before, the estimates for EpipwR are much closer to the true values than the OCplus estimates, particular at higher sample sizes.

**Table 3.** EpipwR and OCplus power calculations against average power from 1000 sub-sampled BMI datasets.^a^

*n*	% of truly significant tests rejected	EpipwR	OCplus
100	0.1224	0.0386	0.0109
200	0.4653	0.4412	0.2395
300	0.7808	0.7946	0.5391
422	0.9367	0.9510	0.7753

a EpipwR parameters were specified to have a maximum margin of error of 0.01.

## 4 Discussion

EpipwR is the first open-source tool specifically designed for sample size determination of EWAS with continuous phenotypes. Similar to many power analysis tools for large-scale studies, it employs simulations to replicate empirical studies. But because these are limited to non-null and a small number of null tests, EpipwR is much faster than existing, fully simulated EWAS power analysis tools (Graw *et al.* 2019). It is important to note that, like all power analysis tools, EpipwR makes several simplifying assumptions, and its outputs should be viewed as preliminary estimates rather than exact calculations.

Although EpipwR is mainly introduced in this manuscript as a power calculator for continuous phenotypes, we also provide a function to calculate power for case-control study designs as well. In the future, the methodology of EpipwR can be expanded to much more sophisticated scenarios, such as the inclusion of one or more confounding variables, estimating correlation across CpG sites that cannot be explained by the phenotype, and more complicated study designs (ANOVA, mixed effect models, etc.).

## Supplementary Material

vbaf150_Supplementary_Data

## Data Availability

All data used in EpipwR is publicly available. See Table 1 for the gene expression omnibus (GEO) identifiers for all data sets.
