# Supplementary material for: EpipwR: efficient power analysis for EWAS with continuous outcomes
Source: Bioinform Adv. 2025 Jun 25;5(1):vbaf150. doi: 10.1093/bioadv/vbaf150 (PMC12303865; doi:10.1093/bioadv/vbaf150)
Supplement: vbaf150_Supplementary_Data [file vbaf150_supplementary_data.zip › File_S1_Supplementary_Material.pdf]

Supplementary Material for EpipwR: Efficient Power Analysis for EWAS with Continuous Outcomes

Jackson Barth and Austin W. Reynolds

Pearson and two-sample test statistic relationship

Recall that the goal is to find a mean difference (δ) that can find an equivalent test statistic with $n-2$ degrees of freedom under an equal variance assumption for a given correlation r. Assume that the split occurs with an equal sample size in each group. The traditional mean difference test statistic then has the following form:

$$\frac{\delta - 0}{\sigma\sqrt{\frac{1}{n/2} + \frac{1}{n/2}}} = \frac{\delta\sqrt{n}}{2\sigma} \sim t_{n-2}$$

We set this equal to the pearson T-statistic and solve for δ:

$$\frac{\delta\sqrt{n}}{2\sigma} = \frac{r\sqrt{n-2}}{\sqrt{1-r^2}} \rightarrow \delta = \frac{2\sigma r}{\sqrt{1-r^2}}\sqrt{\frac{n-2}{n}}$$

note that, when n is large and r is small (which is often the case for EWAS), this has the approximate form $\delta \approx 2\sigma r$. As a test case, assume $r = 0.2, n = 800$, and (WLOG) $\sigma = 1$. The exact Pearson test statistic is

$$\frac{r\sqrt{n-2}}{\sqrt{1-r^2}} = \frac{0.2\sqrt{798}}{\sqrt{1-0.2^2}} = 5.766$$

Converting to the exact δ:

$$\delta = \frac{2(0.2)}{\sqrt{1-0.2^2}}\sqrt{\frac{798}{800}} = 0.4077 \rightarrow TS = \frac{0.4077}{\sqrt{\frac{1}{400} + \frac{1}{400}}} = 5.766$$

Tables and figures

Table S1. User input options for the power calculation functions for continuous (get_power_cont) and binary (get_power_cc) outcomes.

Name	Description	Notes
dm	Expected number of significantly associated CpGs	
Total	Total number of CpGs tested	
n	Sample size(s) at which power is calculated	Accepts a vector of sample sizes
fdr_fwer	family-wise type I error or false discovery rate (FDR)	Depends on use_fdr
rho_mu (cont only)	Average value of ρ for non-null tests	Accepts a vector
rho_sd (cont only)	Standard deviation of ρ for non-null tests	0 implies that ρ_k= rho_mu for non-null tests
delta_mu (cc only)	Average value of δ for non-null tests	Accepts a vector of averages
delta_sd (cc only)	Standard deviation of δ for non-null tests	0 implies that δ_k= delta_mu for non-null tests
n1_prop (cc only)	Proportion of sample size in group 1	Rounds to nearest integer
Tissue	Reference data used to generate β_{ik}	See package for valid options
Nmax	Maximum number of generated datasets	Minimum number is fixed at 20
MOE	Target margin of error for a 95% confidence interval	See section 2.4
test	Type of test utilized	See package for valid options
use_fdr	Indication that fdr_fwer should be treated as FDR	If false, fdr_fwer is treated as FWER
Suppress_updates	If T, removes intermediate status updates	Has no impact on the results

```
> out <- get_power_cont(
dm=500,
Total=100000,
n=c(100,125,150,175,200),
fdr_fwer=.05,
rho_mu=c(0.3,0.35,0.4),
rho_sd=0, Tissue="Saliva",
Nmax=1000,
MOE=.03,
test="pearson",
use_fdr=T,
Suppress_updates=F)
> out


> EpipwR_plot(out)
```

```
sample_size rho_mu avg_power   sd_power  N    se_power
        100   0.30 0.1403333 0.08294258 30 0.015143174
        125   0.30 0.2834091 0.10035896 44 0.015129683
        150   0.30 0.4852500 0.11408804 56 0.015245656
        175   0.30 0.5902963 0.11076639 54 0.015073397
        200   0.30 0.7403871 0.08369535 31 0.015032129
        100   0.35 0.3679444 0.12881474 72 0.015180962
        125   0.35 0.6158689 0.11830236 61 0.015147065
        150   0.35 0.7489565 0.10367534 46 0.015286095
        175   0.35 0.8796000 0.05303266 20 0.011858464
        200   0.35 0.9232000 0.04748141 20 0.010617166
        100   0.40 0.6177113 0.14950251 97 0.015179680
        125   0.40 0.8202667 0.10062044 45 0.014999609
        150   0.40 0.9253000 0.05495750 20 0.012288870
        175   0.40 0.9580000 0.02690334 20 0.006015769
        200   0.40 0.9873000 0.01342856 20 0.003002718
```

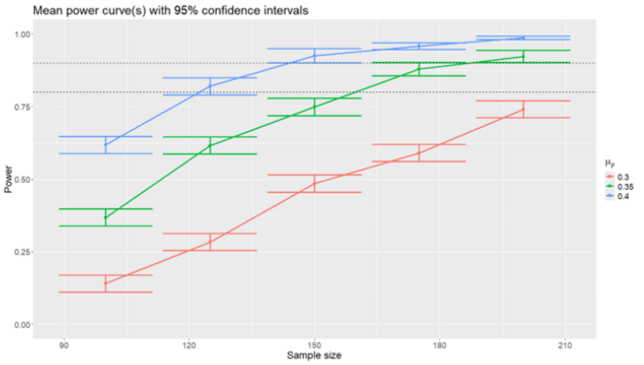

Fig. S1. Code and output of the primary EpipwR functions. The get_power_cont() function produces the R-console table on the right-hand side, while EpipwR_plot() produces the error bar plot.

Table S2. Computation time in seconds of continuous EpipwR for various settings with $K_m = 1000$. All settings shown consider $100k$ total CpG sites and a false discovery rate of 5%. Also reported is the number of simulated data sets used; the algorithm terminates when either (1) $N = 1000$ or (2) $N \geq 20$ and the estimated standard error for power is small enough such that the margin of error for a 95% confidence interval is no bigger than .01. In this way, EpipwR reallocates computational resources away from easily-established results (in the case of $N = 20$, power is typically very close to 0 or 1).

	$\rho = 0.1$	$\rho = 0.3$	$\rho = 0.5$	$\rho = 0.7$	$\rho = 0.9$
$n = 10$	1.32 ($N = 20$)	1.70 (26)	28.39 (452)	62.56 (1000)	1.37 (20)
$n = 50$	4.64 (20)	39.22 (172)	87.37 (382)	4.60 (20)	4.66 (20)
$n = 100$	8.73 (20)	170.20 (393)	8.72 (20)	8.67 (20)	8.72 (20)
$n = 200$	17.49 (20)	69.61 (82)	17.07 (20)	17.02 (20)	17.09 (20)

Table S3. Run time comparison and average margin of error for a 95% confidence interval of power for 3 sample sizes (100, 150, 200) between get_power_cc() and pwrEWAS(). Note that the setting column is formatted as total CpGs (expected significantly associated CpGs). For EpipwR, the MOE argument was set to .005.

Setting	Total Run Time (s)		Average 95% MOE	
	EpipwR	pwrEWAS	EpipwR	pwrEWAS
100k (100)	7.05	636	.005	.015
100k (1k)	9.81	611	.005	0.004
800k (100)	8.90	4,738	.005	0.014
800k (1k)	8.53	4,572	.005	0.004
